# Study of Almond Shell Characteristics

**DOI:** 10.3390/ma11091782

**Published:** 2018-09-19

**Authors:** Xuemin Li, Yinan Liu, Jianxiu Hao, Weihong Wang

**Affiliations:** Key Laboratory of Bio-based Material Science & Technology (Education Ministry), Northeast Forestry University, Harbin 150040, China; 18845632930@163.com (X.L.); liuyinan1983@126.com (Y.L.); JianxiuHao2016@outlook.com (J.H.)

**Keywords:** almond shells, anatomical structure, chemical composition, thermal stability

## Abstract

A large amount of almond shells are disposed of every year. The anatomical and chemical characteristics of almond shells are investigated in this paper in order to contribute to better utilization of these shells. The micromorphology, surface elements, thermal stability, crystallization, chemical composition, and relative properties of almond shells are analyzed. Under observation by microscope and electron microscope, the diameter of almond shells is 300–500 μm for large holes, and 40–60 μm for small holes present in the shell. X-ray photoelectron spectroscopy shows the elements of almond shells include C (72.27%), O (22.88%), N (3.87%), and Si (0.87%). The main chemical constituents of cellulose, hemicellulose and lignin in almond shells account for 38.48%, 28.82% and 29.54%, respectively. The alkaline extract content of almond shells is 14.03%, and benzene alcohol extraction is 8.00%. The benzene alcohol extractives of almond shells mainly contain 17 types of organic compound, including benzene ring, ethylene, carbon three bond, and other mufti-functional groups. Thermal stability analysis shows almond shells mainly lose weight at 260 °C and 335 °C. These characteristics indicate that almond shells have the capacity to be used in composites and absorption materials.

## 1. Introduction

Biomass nutshell is a large yield crop residue which is generally discarded or incinerated. This not only pollutes the environment, but also wastes a large amount of resources. In order to avoid this, many scholars have undertaken research into its potential uses, including the use of biomass nut shells to produce activated carbon. Ahmad et al. used peanut shell adsorption of trichloroethylene for cleaning water [1], while Lan et al. prepared activated carbon from Hawaii nut shells [2]. Martínew et al. prepared activated carbon from walnut shells, and studied its properties [3], and Yang et al. formulated high specific surface area activated carbon from coconut shells by microwave heating [4]. Okutucu et al. produced fungicidal oil and activated carbon from pistachio shells [5]. In addition to the manufacturing of activated carbon, biomass nut shells have a number of other uses. Wongcharee et al. used macadamia nut shell residue as magnetic nanosorbents [6], and Pino et al. studied the biosorption of cadmium using coconut shells [7]. Notably, biomass nut shell has also been used in composites. Alsaadi et al. studied the effect of the content of particles on the mechanical properties of polymer composites [8], and Bae et al. have prepared composite by macadamia nut shell for capturing CO_2_ [9]. Bledzki et al. studied the effects of fiber physics and chemistry on the properties of composites prepared from barley shell and coconut shell reinforced polypropylene [10], and Ayrilmis et al. manufactured chestnut shell reinforced polypropylene composite [11]. In addition, Kasiraman et al. used cashew nut shells as fuel for camphor oil blending [12], and Bartocci et al. proved that biomass shells improve soil health and fertility when used as oil amendment [13]. Wang et al. demonstrated that biocarbon could possibly replace fossil fuel, and help reduce greenhouse gas emissions [14].

Almonds are a type of biomass nut shell and are widely grown in many regions of the world including India, Pakistan, Iran, and China. As of 2014, global output of almonds was three million tons annually (data). Almond shells account for around 35–75% of the total fruit weight, so about 10.5–22.5 million tons of shells were left [15]. This huge quantity of shells has great economic and practical potential, and investigation into the best way to utilize the shell is attracting increasing attention. Rodríguez-Reinoso et al. and Toles et al. prepared activated carbon with almond shells, studying its physical, chemical and adsorptive properties, as well as estimated cost of production [16,17]. Senturk et al. have used almond shells as adsorbents to remove dye rhodamine from aqueous solutions [18]. Mohan et al. prepared magnetic and activated carbon by almond shells to remove 2,4,6-trinitrophenol from water [19], and Essabir et al. reinforced polypropylene with almond shells by extrusion process [20]. Lashgari et al. studied the effect of the loading amount of nano-clay and content of almond shell powder on the strength and properties of polypropylene composites [21].

Due to such studies into almond shell utilization, there is limited knowledge of almond shell structure and chemical properties. Caballero et al. studied the kinetics of slowing the thermal decomposition of almond shells, and analyzed the pyrolysis of lignin and whole cellulose [22]. In the studies of Essabir et al. and Elleuch et al., it is shown that almond shells are absorbent and environmentally friendly [20,23]. This article focuses on the basic chemical composition and physical structure of almond shells in order to make better use of this large quantity bio-resource.

## 2. Materials and Methods

### 2.1. Materials

The almond shells used in this study were purchased from Anhui Liang Zhong Liang Food (Huai’an, China). Poplar wood particles, as a control, were purchased from Jinan Yuanfang Wood Trading Company (Jinan, China). Both almond shells and poplar particles were screened into 40–60 mesh and 60–80 mesh.

### 2.2. Chemical Composition

The main chemical composition of almond shell particles of 40–60 mesh were measured according to Chinese standard GBT 35816-2018 (Standard Method for Determination of Structural Carbohydrates and Lignin in Biomass Raw Material). The content sum of hemicellulose and cellulose was determined by using glacial acetic acid and sodium hypochlorite as a solvent. After repeated washing, the polysaccharide in the almond shells was dissolved, then dried and measured. The content of cellulose was determined by using nitric acid and ethanol as solvent. Other non-cellulosic materials including lignin, hemicellulose, and other materials in the sample are eluted to obtain cellulose. The lignin content was determined by sulfate method. After the sulfuric acid solvent was removed, the residue was measured to determine the quality of lignin.

Particles of 40–60 mesh almond shell were used to measure extractives according to GBT 35818-2018 (Standard Method for Analysis of Forestry Biomass-Determination of Extractives Content). Extractive content of almond shell was measured after the shells were treated with cold water for 48 h, hot water for 6 h, 1% NaOH for 1 h, or benzene alcohol (V benzene/V ethanol = 2:1) for 6 h. In addition, the benzene alcohol extractive solution was diluted to 0.1 mg/mL (V benzene/V ethanol = 2:1), and its chemical composition was measured by GC/MS (7890A-7000B, American Agilent Corporation, Santa Clara, CA, USA).

### 2.3. Infrared Spectrum

Almond shells and poplar particles were scanned by Fourier infrared spectrometer (Nicolette 6700, American Water Corporation, Los Angeles, CA, USA). The KBr disk method was employed for the sample, and wavelength was 500–3500 cm^−1^ at a resolution of 5 cm^−1^.

### 2.4. X-ray Diffraction

Almond shells and poplar particles of 60–80 mesh were measured by X-ray diffraction (D/max-2200VPC, Japan science co., Ltd., Takatsuki City, Osaka, Japan) in the range of 2θ = 5–40° at scanning speed of 5 rad/cm. Two samples were repeated. The X-ray source was a Cu target (Cu Kα = 1.54056), and a nickel filter was used to excite alpha radiation. The crystallization index of the sample was calculated according to the protocol Formula (1) as follows [24]:

(1) $$CrI(\%)=\frac{I_{002}-I_{\mathrm{am}}}{I_{002}}\times 100\%$$

where *I*_002_ is the intensity at 2θ = 22°, and *I*_am_ is the intensity of background scatter at 2θ = 16°.

### 2.5. Surface Element

Almond shells and poplar particles of 60–80 mesh were scanned by an X-ray photo-electron spectrometer (THERMO, American Thermoelectric Group, Waltham, MA, USA). Samples were excited with monochromatic Al Kα rays (1,486.6 eV). The sample binding energy (EB) charge correction was performed using a contaminated carbon C1s (284.8 eV). The X-ray beam was set at 6 mA, and the passing energy was 50 eV. The sputtering ion gun was 1000 eV, and the scanning step was 0.1 eV. Two samples were repeated.

### 2.6. Thermal Stability

The thermal stability of almond shells and poplar particles was measured by high-precision thermo gravimetric analyzer (TG209F1, NETZSCH Scientific Instruments Trading, Bavaria, Germany). The temperature was increased from 0 °C to 800 °C at the rate of 10 °C/min. Two samples were repeated.

### 2.7. Microscopic Morphology

The structure of almond shells was observed by a stereomicroscopic evaluation (JSZ6S, Jiangnan Yongxin Company, Guangzhou, China), and then by scanning electron microscopy (SEM) (QuanTa200, Dutch FEI company, Hillsboro, OR, USA). The cut cross-section of the almond shell was then sprayed with gold and observed at the acceleration voltage of 12.5 kV.

## 3. Results and Discussion

### 3.1. Microscopic Morphology

**Figure 1:**
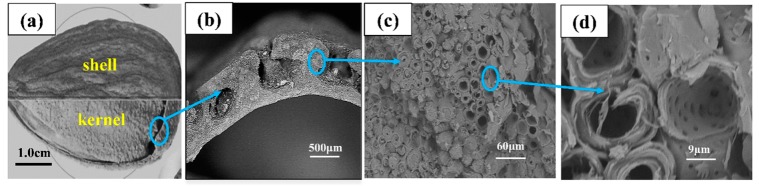
Almond shell micro-topography. (**a**) Overall view; (**b**) Cross-section of shell; (**c**) Dense part of almond shell; (**d**) Hollow ball in the almond shell.

Almond shells have well-developed pore structure, as illustrated in Figure 1, where a row of large holes can be seen distributed in the cross section. Diameter of the big holes is 300–500 μm (Figure 1b). The area around the holes is relatively dense; however, it is full of small hollow balls. Under SEM, almond shells appear to be composed of tightly compacted balls from the view of cross section shown in Figure 1c. The diameter of these hollow balls is about 40–60 μm, and thickness of the ball wall is 20–40 μm. When enlarged 3000 times (Figure 1d), tiny holes are found distributed on the wall of balls, and the wall is visibly layered. The almond shell wall is full of holes of varied diameter, making it light and potentially easily absorbed.

### 3.2. Chemical Composition

The content of cellulose in almond shells is 38.475%, which is lower than that of poplar (containing 44.12% cellulose), but higher than other shells except pistachio shell (Table 1). This indicates that the mechanical properties of composite prepared by almond shells might be higher than that of most biomass nut shells.

Lignin is an amorphous substance without fixed structure; however, it is three-dimensional and highly branched. Lignin generally surrounds microfibers and large fibers. The covalent bond between lignin and polysaccharide greatly enhances the bonding strength between the cellulose fiber and the lignin matrix, and thus plays a role in bonding and strengthening. The lignin content of almond shells is 29.54%, higher than that of poplar, which contains 21.24%. Lignin may be beneficial to improve compatibility with thermoplastic, and it has a certain flame-retardant effect which is important for composites being used as building materials. The special ring structure and hydroxide structure of hemicellulose produces a reactive activity generally higher than that of cellulose. The content of hemicellulose in almond shells and poplar is similar, and higher than other nuts listed in Table 1. This indicates that almond shells may have more prospects for modification.

The extractive content of almond shells and poplar is presented in Table 2. Extractives from alkali method were the highest, followed by benzene and cold water. This result is related to solubility in different solvents. Cold water extractives of almond shells were 3.14%, and poplar was 0.11%. This indicates that almond shells contain more terpenes, phenols, hydrocarbons, and ester compounds than poplar [26]. Terpenes and phenols are the main component of fragrance, thus almond shells could potentially be used as a raw material for creating essence. The content of hot water extract was higher than that of cold water and this is due to the difference in temperature from solubility.

Benzene alcohol extraction of almond shell was 8.00%, a little higher than that of poplar. This indicates that the almond shells and poplar have similar amounts of colored matter content, such as phlobaphene, tannin, fat, wax and resin. The finding shows that there is potential for almond shells to be used in the production of dye ingredients.

The NaOH (1%) extraction of almond shell was 14.03%, and was mainly composed of flavonoids, anthraquinones, phenolic, and lactones. Anthraquinones are antibacterial and have the effects of hemostasis and detoxification, as well as elevating diarrhea and diuresis. Almond shells may also have this function.

The organic compounds contained in benzene alcohol extracts from almond shells were analyzed by mass spectrometry. The results of collecting the compounds with the highest contrast are presented in Table 3. Almond shells mainly contain 17 kinds of organic compounds, with more ring-based compounds, 10 naphthenic hydrocarbons, two lipids, three olefins, two cyclic-olefins, and two kinds of monocyclic aromatic hydrocarbons. This indicates that the shell contains functional groups and active chemical properties. In Table 3, it can be seen that there are more naphthenic hydrocarbons in the almond shells, so it has the potential to be used as a raw material for fuel, or to prepare cosmetics.

### 3.3. Infrared Spectrum

**Figure 2:**
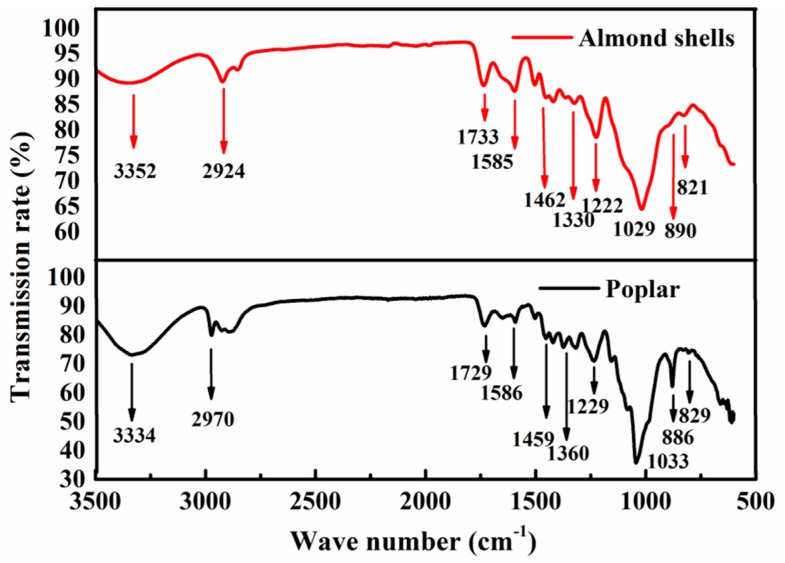
Infrared spectra.

The Fourier-transform infrared (FTIR) spectra of poplar and almond shells seen in Figure 2 and Table 4 are alike, reflecting that they share similar chemical components, which are mainly cellulose, hemicellulose, and lignin. The peak range and sharpness are different and illustrate the content difference of their components. For example, the characteristic peak of 890 cm^−1^ for poplar is sharper than almond shells. This is the characteristic peak of cellulose, indicating that poplar contains more cellulose than almond shells (as Table 1). The characteristic peak at 1580 cm^−1^ and 820 cm^−1^ of almond shells is sharper and stronger than poplar, giving evidence that almond shells contain more lignin than poplar. The characteristic peak at 1730 cm^−1^ of the two samples was similar, indicating that the hemicellulose content is much the same [27].

### 3.4. X-ray Diffraction

**Figure 3:**
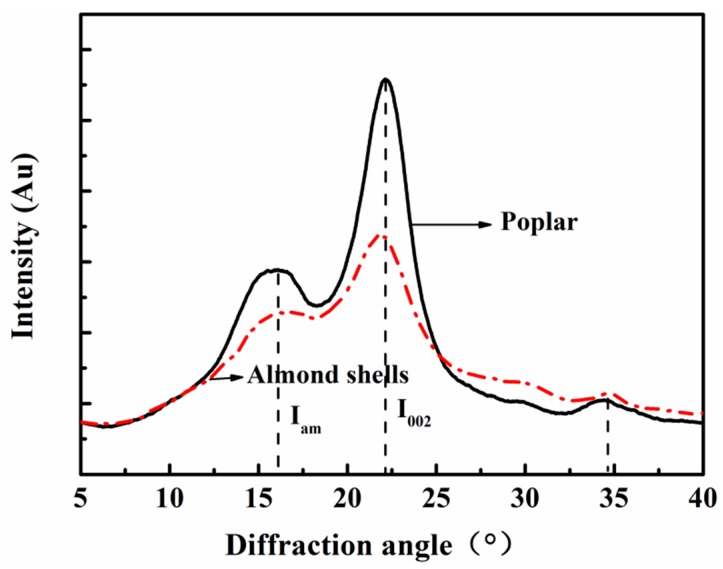
X-ray diffraction spectra.

The X-ray diffraction curves in Figure 3 show that the diffraction peaks of almond shells appeared near 16.6°, 21.48° and 34.67°, indicating its cellulose crystals belong to cellulose I type. In addition, using height method [28] to calculate the crystallization, the crystallization of almond shells is 32.88% lower than that of poplar (48.166%). Crystallization is mainly caused by cellulose, so low crystallization may result in a decrease of mechanical properties. However, compared with other commonly used nuts, crystallization of almond shells is higher, for example, crystallization is 23.25% for chestnut shells, 26.85% for peanut shells, 29.11% for melon shells, 30.61% for macadamia shells, and 23.69% for walnut shells [29]. Combined with cellulose content, almond shells may present higher mechanical properties than other nut shells, which is beneficial in the preparation of composite.

### 3.5. X-ray Photoelectron Spectroscopy Characterization

**Figure 4:**
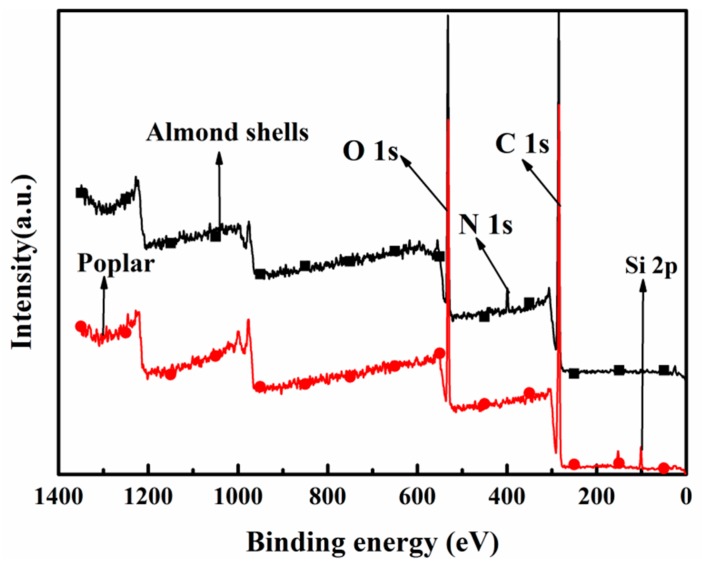
X-ray photoelectron spectroscopy.

As shown in Figure 4, both almond shells and poplar have strong peaks near 532.39 eV/285 eV in X-ray photoelectron spectroscopy, which is the peak of O 1s and C 1s, respectively. In addition, the peak position of N was detected at 398 eV in almond shells, while the peak position of Si at 101.8 eV was detected in poplar. This indicates that there are more N elements in almond shells and Si elements in poplar, and the N may be contained in the protein.

Table 5 shows that the main chemical elements contained in the almond shells are essentially the same as those in other nuts. Compared with the shell of Chinese chestnut, peanut, Sunflower, Hawaii nut, and walnut, almond shells present the highest content of oxygen, and relatively low carbon content. Compared to poplar, nut shells generally contain less silicon and more nitrogen elements. Almond shells might therefore have advantages in fire resistance when preparing composite, due to the flame inhabitant capabilities of N.

**Figure 5:**
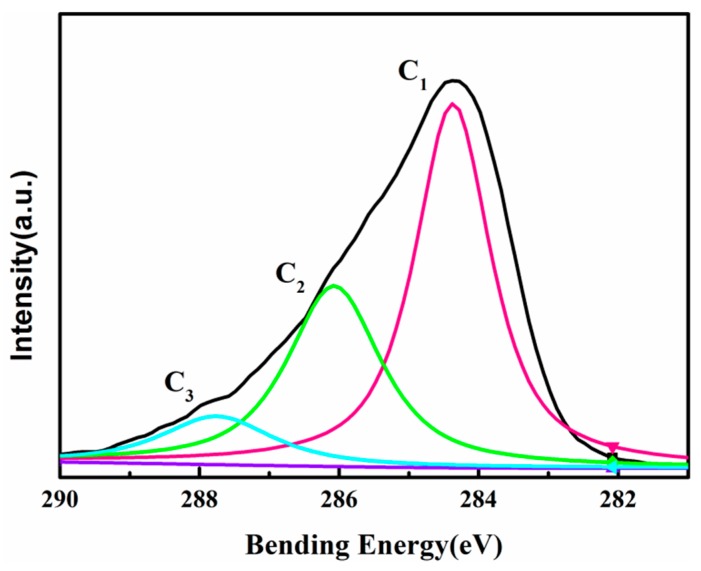
High-resolution C 1s maps of almond shells.

The software XPS Pesk was used to fit the peak of the curve for Figure 5, and C_1_, C_2_, and C_3_ was used to represent the different states of carbon. The main form of carbon in almond shells is C_1_, C_2_, and C_3,_ as listed in Table 6. The characteristic peak of binding energy 284.38 eV was mainly produced by C–H bond in Figure 5. Its electronegativity was small, so the binding energy was small. The characteristic peak of binding energy 286.08 eV was mainly produced by C–OR, in which binding energy was higher. The binding energy of C_3_ (287.79 eV) was mainly produced by C=O.

In Table 6, C_2_ content of almond shells was higher than that of other biomass nut shells, indicating that there are more –OR groups in the almond shells. This result is consistent with Table 1, showing that almond shells were likely to contain more cellulose. The content of C_3_ is lower than that of other biomass nut shells, which illustrates that the C=O in the almond shell was less than that of other biomass nut shells.

### 3.6. Thermal Stability

**Figure 6:**
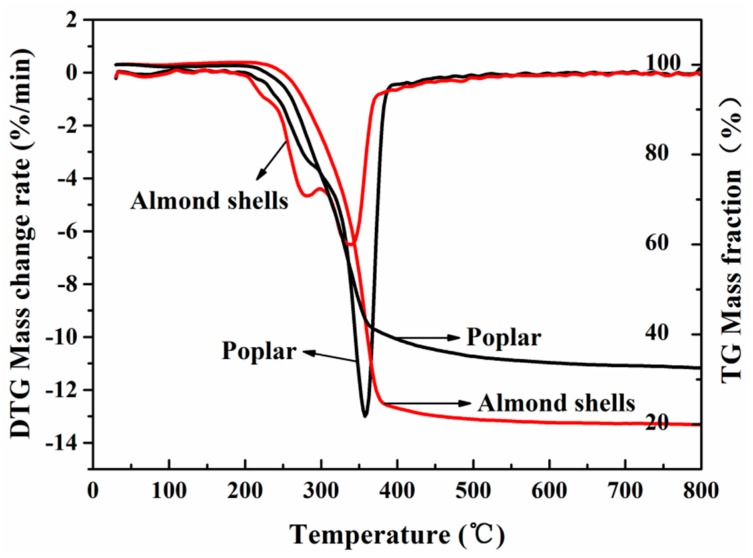
Thermal gravity and derivative thermogravimetric.

The pyrolysis process of almond shells is presented in Figure 6. From room temperature to 200 °C, almond shells and poplar have a small weight loss peak due to the water loss stage. In the range of 200–350 °C, both poplar and almond shells display a weight loss peak which is mainly formed by hemicellulose and lignin. Weight loss of almond shells occurred earlier than poplar, however, and the peak of the almond shells was sharper than poplar. The highest loss peaks of poplar and almond shells appeared at 300–500 °C. The poplar had the highest peak at 350 °C, while the almond shell peak was at 338 °C. Poplar’s peak is wider and sharper than almond shells. Thermal interpretation of cellulose and lignin is formed by volatiles, mainly relying on the pyrolysis of cellulose [30]. The higher peaks of poplar indicate that it contains more cellulose.

The carbonization phase occurs after 500 °C and pyrolysis is basically completed at this stage. Thus, the curve of nutrient biomass after 500 °C tends to be gentle. In conclusion, the thermal stability of poplar is superior to that of almond shells, but compared with other nut shells, the thermal stability of almond shells is better, for example, the highest pyrolysis temperature of pistachio shells is 337 °C, and cashew nut shells is 318 °C, which is lower than the almond shells (338 °C).

## 4. Conclusions

In this study, the physical structure and chemical properties of almond shells were determined. The following conclusions were obtained.

1. Observed by microscope and electron microscope, almond shells have the diameter of 300–500 μm for large holes, and 40–60 μm for small holes.
2. The elements of almond shells include C (72.27%), O (22.88%), N (3.87%), and Si (0.87%). The main chemical constituents of cellulose, hemicellulose, and lignin account for 38.48%, 28.82% and 29.54%.
3. The alkaline extract content of almond shells was 14.03%, and benzene alcohol extraction was 8.00%. The benzene alcohol extractives of almond shells mainly contain 17 types of organic compound including benzene ring, methylene, carbon three bond, and other multi-functional groups.
4. Thermogravimetric analysis shows almond shells mainly lose weight at 260 °C and 335 °C. This shows that its pyrolysis temperature is lower than poplar; however, it is higher than pistachio shells and cashew nut shells.
5. X-ray diffraction spectra showed that diffraction peaks of almond shells appeared near 16.6°, 21.48° and 34.67°. Cellulose crystals belong to cellulose I type. The crystallization of almond shells is 32.88%, which is lower than poplar; however, it is higher than chestnut shells, peanut shells, melon shells, macadamia shells, and walnut shells.
6. X-ray photoelectron spectroscopy showed that almond shells contained more N than poplar and five other kinds of nut shell (chestnut shells, peanut shells, sunflower shells, Hawaii nut shells, and walnut shells). There was less Si in the almond shells compared to poplar.

## Figures and Tables

**Table 1:** Proportion of cellulose, hemicellulose, lignin in six kinds of biomass (wt %).

Sample	Cellulose	Hemicellulose	Lignin
Almond shells	38.47 ± 0.39	28.82 ± 0.25	29.54 ± 0.11
Poplar	44.12 ± 0.23	30.21 ± 0.11	21.24 ± 0.31
Coconut shells	34.12 ± 0.20	22.36 ± 1.47	28.04 ± 0.57
Walnut shells	36.38 ± 0.05	27.85 ± 0.31	43.70 ± 0.57
Chestnut shells	21.47 ± 0.27	16.28 ± 0.35	36.58 ± 0.26
Pistachio shells	43.08 ± 0.19	25.30 ± 0.46	16.33 ± 0.41

Note: The data of almond shells and poplar were measured by authors, and the rest of the measurements are derived from literature [25].

**Table 2:** Extractive content of different extraction methods (wt %).

Extraction Method	Almond Shells Extractive Content	Poplar Extractive Content
Cold water extraction	3.14	0.11
Hot water extraction	4.64	8.64
NaOH (1%) extraction	14.03	20.56
Benzene alcohol extraction	8.00	7.50

**Table 3:** Compound shell containing extracts of almond shells.

Chemical Compound	Molecular Formula	Structural Formula	Chemical Compound	Molecular Formula	Structural Formula
Cyclopentane, ethyl-	C_7_H_14_	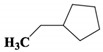	Cyclohexane,1,2-dimethyl-, *cis*-	C_8_H_16_	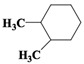
Toluene	C_7_H_8_	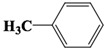	Cyclohexane, ethyl-	C_8_H_16_	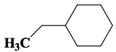
Cyclohexane, 1,3-dimethyl-, *cis*-	C_8_H_16_	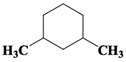	Cyclohexane, 1,1,3-trimethyl	C_9_H_18_	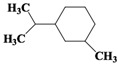
Cyclohexane, 1,1-dimethyl-	C_8_H_16_	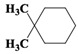	Ethylbenzene	C_8_H_10_	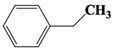
Cyclopentane,1-ethyl-3-methyl-	C_8_H_16_	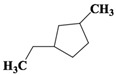	*Cis*-bicyclo[4.2.0]octane	C_8_H_14_	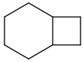
(Z)-Hex-3-enyl (E)-2-methylbut-2-enoate	C_11_H_18_O_2_	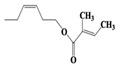	Hexane,3-methyl-4-methylene-	C_8_H_16_	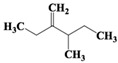
Cyclohexane,1,3-dimethyl-, *cis*-	C_8_H_16_	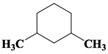	Cyclohexane, ethenyl-	C_8_H_14_	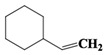
Butanoicacid,2-methyl-,1,2-dimethylpropyl ester	C_10_H_20_O_2_	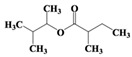	Cyclopentane, (1-methylethyl)-	C_8_H_16_	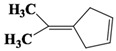
Cyclohexane,1,4-dimethyl-	C_8_H_16_	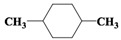			

**Table 4:** Absorption band assignment in the infrared spectrum of almond shell and poplar.

Wavenumber (cm^−1^)	Functional Group	Vibration Type	Cause
3300~3500	–OH	stretching vibration	cellulose, hemicellulose
2900~2935	–CH	stretching vibration	-
1640~1735	C=O	stretching vibration	lignin, hemicellulose
1580~1605	benzene ring	stretching vibration	lignin
1455~1465	–CH_3_O	stretching vibration	lignin
1320~1430	–CH	bending vibration	-
1221~1230	C–C C–O	stretching vibration	lignin
1025~1035	C–O	stretching vibration	cellulose, hemicellulose, and lignin
885~895	R_2_C=CH_2_	bending vibration	-
810~833	benzene ring	disubstituted benzene	-

**Table 5:** Surface chemical composition and relative content of nut and poplar (wt %).

Sample Type	n _C_	n _O_	n s_i_	n _N_
Chestnut shells	80.26	17.28	0.85	1.61
Peanut shells	74.16	21.72	0.34	3.78
Sunflower shells	78.86	18.13	0.42	2.59
Hawaii nut shells	79.16	18.92	0.26	1.67
Walnut shell	78.84	19.32	0.16	1.67
Poplar	74.56	20.93	4.51	-
Almond shells	72.27	22.88	0.87	3.87

Note: The data of almond shells are measured by authors, and the rest are derived from reference [29].

**Table 6:** X-ray photoelectron spectroscopy (XPS) test results of almond shells C 1s.

Sample	Binding Energy (eV)			A (%)		
	C_1_ 1s	C_2_ 1s	C_3_ 1s	C_1_ 1s	C_2_ 1s	C_3_ 1s
Almond shells	284.38	286.08	287.79	55.88	32.35	11.76
Chestnut shells	284.8	286.28	286.93	63.18	17.70	19.12
Peanut shells	284.8	286.24	286.79	48.93	16.34	34.73
Sunflower shells	284.8	286.25	288.07	61.99	25.94	12.07
Hawaii nut shells	284.8	286.25	287.70	54.58	30.85	14.57
Walnut shells	284.8	286.25	287.10	55.22	23.17	21.61

Note: The data of almond shells are measured by authors, the rest of the data is derived from reference [29].
